# Uptake of N_2_O_5_ by aqueous aerosol unveiled using chemically accurate many-body potentials

**DOI:** 10.1038/s41467-022-28697-8

**Published:** 2022-03-10

**Authors:** Vinícius Wilian D. Cruzeiro, Mirza Galib, David T. Limmer, Andreas W. Götz

**Affiliations:** 1 San Diego Supercomputer Center, University of California San Diego, La Jolla, CA 92093 USA; 2 Department of Chemistry and Biochemistry, University of California San Diego, La Jolla, CA 92093 USA; 3 Department of Chemistry, University of California, Berkeley, CA USA; 4 Kavli Energy NanoScience Institute, Berkeley, CA USA; 5 Materials Science Division, Lawrence Berkeley National Laboratory, Berkeley, CA USA; 6 Chemical Science Division, Lawrence Berkeley National Laboratory, Berkeley, CA USA

**Keywords:** Atmospheric chemistry, Molecular dynamics, Statistical mechanics, Chemical physics, Reaction kinetics and dynamics

## Abstract

The reactive uptake of N_2_O_5_ to aqueous aerosol is a major loss channel for nitrogen oxides in the troposphere. Despite its importance, a quantitative picture of the uptake mechanism is missing. Here we use molecular dynamics simulations with a data-driven many-body model of coupled-cluster accuracy to quantify thermodynamics and kinetics of solvation and adsorption of N_2_O_5_ in water. The free energy profile highlights that N_2_O_5_ is selectively adsorbed to the liquid–vapor interface and weakly solvated. Accommodation into bulk water occurs slowly, competing with evaporation upon adsorption from gas phase. Leveraging the quantitative accuracy of the model, we parameterize and solve a reaction–diffusion equation to determine hydrolysis rates consistent with experimental observations. We find a short reaction–diffusion length, indicating that the uptake is dominated by interfacial features. The parameters deduced here, including solubility, accommodation coefficient, and hydrolysis rate, afford a foundation for which to consider the reactive loss of N_2_O_5_ in more complex solutions.

## Abstract (web summary)

The reactive uptake of N_2_O_5_ to aqueous aerosol is a major loss channel for nitrogen oxides in the troposphere. Here authors report a theoretical investigation on the N_2_O_5_ uptake into aqueous aerosol and determine the hydrolysis rates by numerically solving a molecularly detailed reaction–diffusion equation.

## Introduction

The uptake of trace gases from the air into aerosol particles impacts a wide range of environmental systems^1,2^. Among other things, such multiphase processes help to determine the oxidative power of the atmosphere by acting as sinks for nitrogen oxides^3,4^. Of particular long-standing interest is the reactive uptake of N_2_O_5_ in aqueous aerosol, which is estimated to account for 15–50% of the loss of NO_*x*_ in the troposphere^5,6^. Despite the significant study, basic questions remain concerning the mechanism of N_2_O_5_ uptake^7–13^. Molecular dynamics simulations can be used to obtain a molecular perspective on gaseous uptake, free of underlying rate limitation assumptions^14^. However, studying such processes theoretically imposes challenges, since uptake coefficients are exponentially sensitive to free energy differences and the simulations involve large systems and long times to model the complex dynamics. While qualitative predictions of mechanisms can be typically studied with conventional empirical force fields or density functional theory-based models^13,15^, quantitative predictions require higher levels of accuracy. To address this challenge, a many-body potential, MB-nrg^16^, has recently been parameterized from coupled-cluster calculations, providing the capability of making quantitative predictions of the thermodynamics and kinetics leading to the N_2_O_5_ uptake.

Gaseous uptake into fluid particles couples thermodynamic constraints of solubility with kinetic details of reaction and diffusion. As a complete analytical analysis of the appropriate reaction–diffusion equations is not typically tenable, approximate models are commonly postulated employing a small number of thermodynamic and kinetic properties^17^. For example, the uptake of N_2_O_5_ in aqueous aerosol has been assumed to follow such a model, determined by bulk accommodation followed by bulk phase hydrolysis and parameterized by its bulk solubility and hydrolysis rate^18^. Such kinetic models typically lack molecular details, neglecting the finite width of the liquid–vapor interface and its potential unique properties. With molecular dynamics simulations these assumptions can be relaxed, and the relevant parameters extracted to inform an atomistic kinetic model^19–21^. Further, by solving the reaction–diffusion equations numerically, the simplified models can be refined.

The validity of the traditional resistor model for the reactive uptake of N_2_O_5_ has been recently called into question due to the difficulty of reconciling the kinetics with field measurements, combined with theoretical work providing indications of interfacial stability and reactivity^15,22^. The mechanism of uptake has been recently explored directly using a neural network-based reactive model, and it was found that interfacial rather than bulk phase processes dictate the observed uptake coefficient^13^. Using training data obtained from density functional theory, this study found that the hydrolysis rate was sufficiently fast at the interface that bulk phase partitioning cannot kinetically compete, and the uptake was determined by a competition between interfacial hydrolysis and evaporation. These calculations found modest agreement with experimental uptake coefficient values, consistent with the expected qualitative accuracy of the model employed. As direct experimental confirmation of the importance of the interface is difficult, an alternative means of validating it is to employ models with higher chemical accuracy. This is the aim of the current work, to apply a quantitatively accurate potential to extract the thermodynamic and kinetic properties underpinning the uptake of N_2_O_5_ into water.

MB-nrg potentials can serve to make a quantitative prediction of gaseous uptake as they can be accurate yet computationally amenable to the large system sizes and long timescales required to simulate interfacial processes just like the MB-pol water model^23,24^. Contrary to common neural network models, these many-body potentials have an explicit representation for long-range interactions, which can be important at extended interfaces^25^. For example, it has been shown that MB-pol^26,27^ yields quantitative accuracy for a variety of molecular properties across water’s phase diagram^26–43^ including at the water-vapor interface^44^. Extensions of this modeling framework to describe mono-atomic ions and small molecules in aqueous solutions as well as generic mixtures of molecules have been recently realized^45–49^. These MB-nrg models include a model of N_2_O_5_ that has been developed using analogous approaches^16^. This MB-nrg model was demonstrated to yield comparable accuracy with respect to the coupled cluster reference data it was parameterized on, enabling highly accurate simulations of N_2_O_5_ in aqueous environments. While not able to describe reactions with water, the model nevertheless is capable of quantifying the processes that establish the physical uptake of N_2_O_5_.

Here we employ this MB-nrg model to study the physical uptake of N_2_O_5_ into water using molecular dynamics simulations and enhanced sampling techniques, making quantitative predictions of the thermodynamics and kinetics of N_2_O_5_ uptake. We subsequently leverage the quantitative accuracy of the model to parameterize and solve a reaction–diffusion equation and infer hydrolysis rates consistent with experiment, providing a complete quantitative picture of the reactive uptake of N_2_O_5_ by aqueous aerosol. We find a short reaction–diffusion length, indicating that the uptake is dominated by interfacial features in the vicinity of the liquid/vapor interface.

## RESULTS

**Fig. 1:**
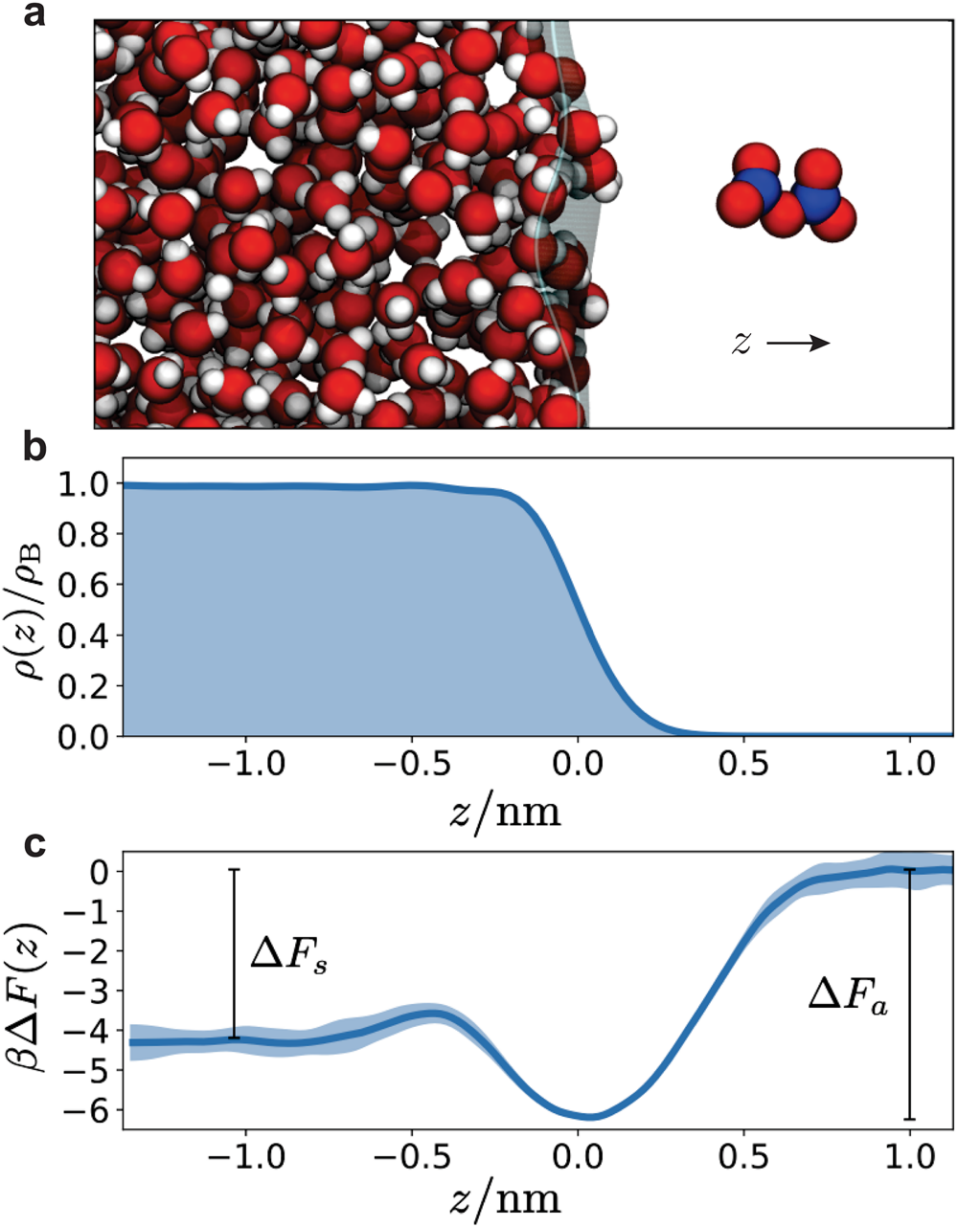
Thermodynamics of N_2_O_5_ solvation. **a** Characteristic snapshot of N_2_O_5_ near the water–vapor interface. **b** Water density profile where *z* = 0 demarks the Gibbs dividing interface and *ρ*_B_ is the bulk water density. **c** Free energy to move N_2_O_5_ in the *z* direction, where the shaded region are one standard deviation error bars. Δ*F*_a_ and Δ*F*_s_ are the free energies of adsorption and solvation, respectively.

In order to extract the thermodynamic and kinetic properties that determine the uptake of N_2_O_5_, we have simulated a system containing a slab of liquid water in contact with its vapor and a single N_2_O_5_ molecule as described in the Methods section and illustrated in Fig. 1a. The corresponding density profile of water along the direction perpendicular to the interface, *ρ*(*z*), is shown in Fig. 1b, exhibiting the expected sinusoidal profile consistent with emergent capillary waves^50^. The bulk density of the MB-pol model *ρ*_B_ is 1.007 g/cm^3^ ^29^. We take the origin of *z* to be coincident with the Gibbs dividing surface of the interface.

### Thermodynamics of adsorption and solvation

Using this simulation setup, we first considered the thermodynamics of N_2_O_5_ solvation in liquid water. Figure 1c shows the free energy profile for moving a gaseous N_2_O_5_ into liquid water. Supplementary Fig. 1 depicts the free energy profile in more detail and the Supplementary Notes and Supplementary Table 1 contain an overview of all thermodynamic and kinetic parameters discussed in this text. For both *z* ≫ 0 and *z* ≪ 0, the free energy profile is flat, reflecting the translationally invariant bulk liquid and vapor on either side of the interface. We define the offset between these asymptotic values as *β*Δ*F*_s_ = −4.3 ± 0.1, the solvation free energy for the gas phase N_2_O_5_, where *β* = 1/*k*_B_*T* and *k*_B_ is Boltzmann’s constant. In between these two extremes, the free energy is non-monotonic and exhibits a global minimum approximately centered at the Gibb’s dividing surface and a barrier to move the N_2_O_5_ molecule from this interfacial position into the bulk liquid. Relative to the gas phase, the global minimum corresponds to an interfacial adsorption free energy of *β*Δ*F*_a_ = −6.2 ± 0.1. The interfacial adsorption indicates N_2_O_5_ is relatively hydrophobic, consistent with previous observations of its relatively weak solvation^13,15,51^. This free energy profile dictates that the equilibrium density profile of N_2_O_5_ would be inhomogeneous in the vicinity of the liquid–vapor interface, a feature neglected in typical kinetic models.

### Solubility

From the free energy profile we can calculate the solubility of N_2_O_5_ in liquid water. In dilute solution at concentration *c*_*l*_ in contact with a solute with partial pressure *p*, this solubility is traditionally reported as a Henry’s law constant defined as^52^

1 $$H=\frac{{c}_{l}}{p}=\beta {{e}}^{-\beta {{\Delta }}{F}_{{{{{{{{\rm{s}}}}}}}}}}$$

and computable from the solvation free energy defined operationally from our free energy profile. This estimate gives a Henry’s law constant *H* = (3.0 ± 0.4) M/atm. This value is in line with typical inferences from experiment, which range between 1 and 10 M/atm, though its direct measurement is hindered by the facile hydrolysis of N_2_O_5_^52,53^. This value is higher than recent estimates employing fixed charge force fields and neural network potentials, each of which found a value closer to 0.5 M/atm^13,15^.

### Diffusion in bulk and at the liquid/vapor interface

As gaseous uptake couples thermodynamics and kinetics, we have also characterized the dynamical processes of N_2_O_5_ as it moves between phases across the liquid–vapor interface. Before considering the rare events of evaporation and solvation, we first discuss the diffusive properties of N_2_O_5_ in the bulk liquid. With a simulation of N_2_O_5_ immersed in a bulk liquid containing 272 water molecules, we have estimated the self-diffusivity of N_2_O_5_ by computing mean-squared displacements. The value obtained for the self-diffusion constant of N_2_O_5_ derived from the average mean-squared displacement was (1.53 ± 0.06) × 10^−5^ cm^2^/s. Hydrodynamic effects are known to suppress the diffusion constant for finite systems employing periodic boundary conditions^54^. Using the known experimental viscosity of liquid water at ambient conditions^29^, we can correct for these finite size effects resulting in a diffusion constant in the thermodynamic limit of (1.89 ± 0.06) × 10^−5^ cm^2^/s. We have also estimated the change in the diffusion constant at the liquid–vapor interface^55^, and find an increase from its bulk value to (5.3 ± 0.1) × 10^−5^ cm^2^/s.

### Adsorption and evaporation rates

Evaporation from the liquid–vapor interface and solvation into the bulk are both activated processes with barriers estimated from the free energy in Fig. 1 to be larger than typical thermal values. As such, they are rare events and difficult to sample with straightforward simulations. However, as we consider a system whose dynamics satisfies detailed balance, we can alternatively study comparatively typical events like desolvation and adsorption, and infer their reverse using the previously evaluated free energy profile^56^. Definitions for these different dynamical processes are well described in ref. ^17^.

**Fig. 2:**
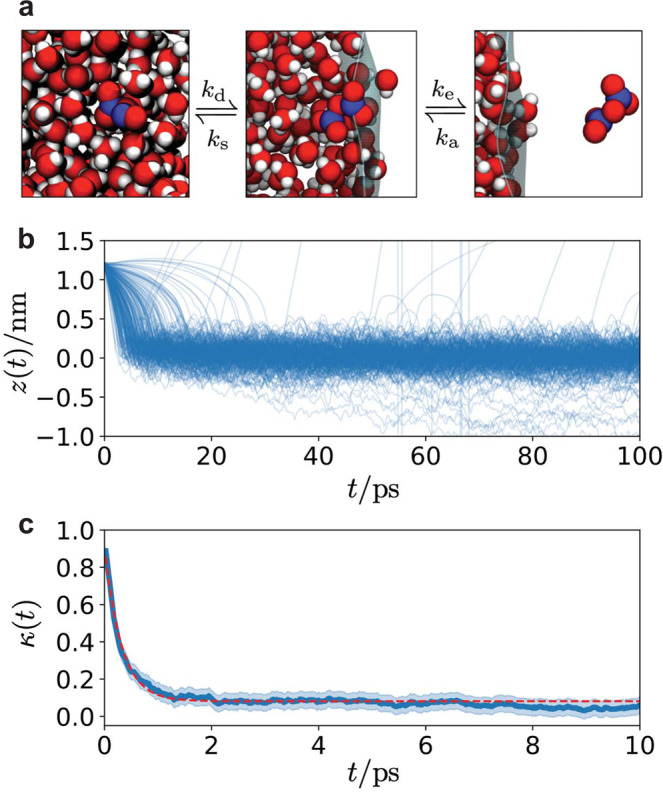
Kinetics of N_2_O_5_ adsorption and solvation. **a** Snapshots of N_2_O_5_. Adsorption and evaporation takes N_2_O_5_ between the vapor (right) and interface (center), while solvation and desolvation takes it between the interface and the bulk (left), with corresponding rate constants. **b** Scattering trajectories following the *z* component of the center of mass of N_2_O_5_. **c** Transmission coefficient for transitions between the liquid and the interface. The shaded region are one standard deviation error bars. The solid red line is an exponential fit.

To compute the adsorption and therefore evaporation rates, we have sampled 250 scattering trajectories whereby an initially gas phase N_2_O_5_ placed at *z* = 1.2 nm, shown in the right panel of Fig. 2a, is evolved toward the liquid slab. To do this we take 10 distinct equilibrium configurations of N_2_O_5_ generated by constraining its center of mass to *z* = 1.2 nm, and draw 25 realizations of a Maxwell–Boltzmann distributed velocity at 300 K for each. Figure 2b reports the trajectories of the center of mass of the N_2_O_5_ as it impinges on the liquid slab. Overwhelmingly, the incipient gas phase N_2_O_5_ molecule meets the interface and sticks, with only 11 out of the 250 scattering trajectories exhibiting a back scattering event, with N_2_O_5_ bouncing off of the interface and going back into the gas phase within the 100 ps observation time employed. The scattering rate is quantified with the so-called thermal accommodation coefficient, *S* = 0.96 ± 0.06, relating the probability of being accommodated at the interface upon collision, consistent with previous simulations^15^. This is likely a lower bound as some of the back scattering events can be attributable to equilibration at the interface followed by subsequent evaporation. The near unity value implies a lack of a barrier to adsorption, that subsequent evaporation is analogously limited only by the free energy of adsorption, and that uptake is not significantly influenced by this initial thermal accommodation. The corresponding rates of adsorption, *k*_a_, and evaporation, *k*_e_, can be computed from kinetic theory^17^. Specifically, the rates are given by

2 $${k}_{{{{{{{{\rm{a}}}}}}}}}=S\frac{v}{4}\,,\quad {k}_{{{{{{{{\rm{e}}}}}}}}}={k}_{{{{{{{{\rm{a}}}}}}}}}{{e}}^{\beta {{\Delta }}{F}_{{{{{{{{\rm{a}}}}}}}}}}$$

where $v=\sqrt{8/\beta \pi m}$ is the average molecular speed of N_2_O_5_. These are *k*_a_ = 57 nm/ns and *k*_e_ = 0.11 nm/ns.

### Solvation and desolvation rates

We have also computed the rates of solvation and desolvation following the Bennet-Chandler approach^57^. Identifying *z*^†^ = −0.42 nm as the location of the putative transition state for solvation into the bulk liquid from the interface (see Fig. 1c), we can estimate the rate of solvation and analogously desolvation by computing the transmission coefficient, *κ*, for committing to the interface conditioned on starting at the transition state. The transmission coefficient is defined as^57^

3 $$\kappa (t)=\frac{{\langle v(0){{\Theta }}(z(t)-{z}^{{{{\dagger}}} })\rangle }_{{z}^{{{{\dagger}}} }}}{\langle | v| \rangle /2}$$

where Θ is the step function and the brackets denote an ensemble average where in the numerator it is conditioned on starting at the transition state. We have evaluated the transmission coefficient using 2000 trajectories. Like the scattering calculations, we have taken 80 different equilibrium configurations of N_2_O_5_ at *z* = *z*^†^, and compose 25 Maxwell–Boltzmann velocity distributions at 300 K for each. Each trajectory was evolved for 10 ps. Figure 2c shows that *κ* decays to 0.08 over 1 ps, consistent with a diffusive barrier crossing. From the plateau, we can estimate the rates to solvate into the bulk, *k*_s_, and desolvate into the interface, *k*_d_, as

4 $${k}_{{{{{{{{\rm{d}}}}}}}}}=\kappa \frac{v}{2\ell }{{e}}^{\beta {{\Delta }}{F}_{{{{{{{{\rm{b}}}}}}}}}}\,,\quad {k}_{{{{{{{{\rm{s}}}}}}}}}={k}_{{{{{{{{\rm{d}}}}}}}}}{{e}}^{\beta ({{\Delta }}{F}_{{{{{{{{\rm{a}}}}}}}}}-{{\Delta }}{F}_{{{{{{{{\rm{s}}}}}}}}})}$$

where *β*Δ*F*_b_ = 0.8 is the barrier to move from the bulk liquid to the interface and *ℓ* = 0.6 nm is the width of the interface. The width of the interface is determined by fitting the free energy minima to a parabola and integrating the resultant Gaussian distribution from 1 nm > *z* > *z*^†^. We find *k*_d_ = 340/ns and *k*_s_ = 51/ns.

### Mass accomodation

We can also evaluate the mass accomodation coefficient *α*, defined as the probability of a gas molecule striking the liquid surface to solvate into the bulk liquid phase in absence of surface reactions^17^. This fundamental parameter determines the transfer rate of N_2_O_5_ across the surface into the bulk liquid and can be computed from the sticking coefficient *S* and the free energy profile as

5 $$\alpha =\frac{S}{1+{{e}}^{\beta ({{\Delta }}{F}_{{{{{{{{\rm{s}}}}}}}}}+{{\Delta }}{F}_{{{{{{{{\rm{b}}}}}}}}})}}$$

We find *α* = 0.93 ± 0.06, in agreement with experiments that infer a value larger than 0.4 for N_2_O_5_^58^.

### Reactive uptake through interfacial and bulk hydrolysis

Experimentally, N_2_O_5_ undergoes facile irreversible hydrolysis with water and this reaction ultimately determines the reactive uptake in aqueous aerosol. While we cannot simulate the reactive event with the MB-nrg potential employed directly, we can still make an inference into the reactive uptake. Using the thermodynamic and kinetic parameters evaluated from the molecular dynamics simulations, we can parameterize a molecularly detailed reaction–diffusion equation. Specifically, we consider the diffusive dynamics accompanying an initially adsorbed N_2_O_5_ molecule as it enters the bulk liquid or evaporates, and address what would happen if it were able to also undergo hydrolysis.

Consistent with the near unity thermal accommodation *S*, we assume an initially adsorbed molecule locally equilibrated at the interface. The subsequent evolution of its concentration profile, *c*(*z*, *t*), can be solved for using a Smoluchowski equation^59^ of the form,

6 $$\frac{\partial c(z,t)}{\partial t}=\frac{\partial }{\partial z}D(z){{e}}^{-\beta {{\Delta }}F(z)}\frac{\partial }{\partial z}{{e}}^{\beta {{\Delta }}F(z)}c(z,t)-{k}_{{{{\rm{h}}}}}(z)c(z,t)$$

where Δ*F*(*z*) is the free energy profile from Fig. 1c, *D*(*z*) is the diffusion constant, and *k*_h_(*z*) is the unknown hydrolysis rate, both of which in principle vary through space^60,61^. The first term is a drift diffusion encoding the stationary distribution implied by the free energy profile, while the second accounts for loss due to reaction. In practice, we fit the free energy to an analytic function form, $\beta {{\Delta }}F(z)={a}_{1}\tanh [(z-{a}_{2})/{a}_{3}]-{a}_{4}\exp [-{(z-{a}_{5})}^{2}/{a}_{6}]\;+{a}_{7}\exp [-{(z-{a}_{8})}^{2}/{a}_{9}]$. Equation (6) is valid only for the liquid and interface, not the vapor, since it is overdamped^59^. In order to model the vapor, we employ absorbing boundary conditions *c*(*z* = 1 nm, *t*) = 0, and consider a domain that extends deeply enough into the liquid that the results are insensitive to the reflecting boundary condition employed there, ∂_*z*_*c*(*z* = −30 nm, *t*) = 0. We solve Eq. (6) with a normalized Gaussian initial condition, localized in the free energy minima near the Gibbs dividing surface where a standard deviation of 0.05 nm was found to well approximate the curvature of the interfacial minima. In practice, we employ a simple finite difference scheme with constant grid spacing of 0.02 nm and timestep 0.018 ps.

**Fig. 3:**
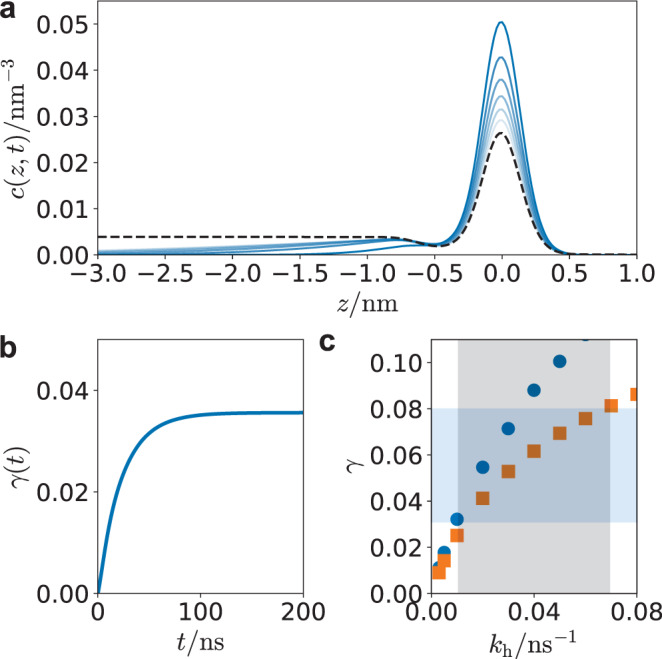
Reactive uptake from the reaction–diffusion equation. **a** Relaxation of the initial concentration profile. Blue lines are *c*(*z*, *t*) separated by 0.25 ns and the dashed black line is the equilibrium profile computed from $\exp [-\beta {{\Delta }}F(z)]$. **b** An example time-dependent reactive uptake coefficient *γ*(*t*). Both a) and b) is computed with *k*_h_ = 0.02 ns^−1^ and the interfacial rate equal to *k*_h_/5. **c** Asymptotic uptake coefficients without (orange squares) and with (blue circles) interfacial reactivity. Blue regions denote the range of uptake coefficients observed experimentally on pure water, and gray the corresponding likely range of bulk hydrolysis rates.

In the absence of any reactions, an initial interfacial concentration of N_2_O_5_ will relax through a competition between diffusion into the bulk liquid and evaporation into the vapor towards the steady-state determined by the free energy profile. Figure 3a illustrates the relaxation of this concentration profile. The initial Gaussian distribution quickly looses amplitude and a diffusive front propagates into the bulk liquid, while concentration is irreversibly lost to the vapor. The initial rates to evaporate and solvate are consistent with our explicit molecular simulation calculations. In the presence of hydrolysis, in addition to loss from evaporation, there can be loss due to reaction. Since the concentration is normalized to 1, the overall reactive uptake, *γ*, can be computed by the portion of the loss through the reactive channel,

7 $$\gamma (t)=\int\nolimits_{0}^{t}{{{{{\rm{d}}}}}}t^{\prime} \int {{{{{\rm{d}}}}}}z\,{k}_{{{{{{{{\rm{h}}}}}}}}}(z)c(z,t^{\prime} )$$

We model the hydrolysis rate as having two characteristic values, one in the bulk for *z* < −0.5 nm, denoted *k*_h_, and one at the interface for −0.5 < *z* < 0.5 nm taken to be a fraction of the bulk value, while it is set to zero in the vapor for *z* > 0.5 nm. The interfacial region was determined from the inflection points in the free energy curve and numerical tests have shown that the results are insensitive to the precise width of the interfacial region. An example time series for the reactive uptake is shown in Fig. 3b, which rises from 0 to a plateau value at times much longer than the characteristic time associated with the bulk hydrolysis rate. This asymptotic value is the reactive uptake coefficient.

The reactive uptake as a function of the bulk hydrolysis rate is shown in Fig. 3c. For each bulk hydrolysis rate, we have computed *γ* setting the interfacial rate equal to the bulk value, and also setting it to zero. We believe these are the two likely extremes, as previous explicit calculations found that interfacial hydrolysis was suppressed relative to the bulk^13^. Experimentally, the range of reactive uptake coefficients on pure water has been reported between 0.03 and 0.08^7–9^, which is consistent with a bulk hydrolysis rate between 0.01 and 0.07 ns^−1^ in Fig. 3c. These rates are slower than those computed directly from a previous neural network model (0.2 ns^−1^)^13^, but faster than that typically inferred experimentally (0.002 ns^−1^)^62^. The disagreement with respect to the neural network model could likely be a failure of the density functional used in the training data, by delocalizing the charge transfer accompanying hydrolysis^63–65^. The disagreement with the rates inferred experimentally is because those are based on reactive uptake models that neglect interfacial reactivity and stability. The confirmation of the importance of the interface, reducing the diffusion into the bulk and accounting for a significant fraction of hydrolysis, agrees with the previous neural network model study^13^, and the need to revise the standard resistor model. The hydrolysis rate obtained with our modeling in addition to the other parameters relevant to reactive uptake are summarized in Table 1.

**Table 1:** Physical and chemical properties of N_2_O_5_.

Henry’s law constant	*H*	(3.0 ± 0.4) M/atm
Diffusivity	*D*	(1.89 ± 0.06) × 10^−5^ cm^2^/s
Sticking coefficient	*S*	0.96 ± 0.06
Mass accommodation	*α*	0.93 ± 0.06
Hydrolysis rate^*a*^	*k*_h_	(4 ± 3) × 10^−2^ ns^−1^

^a^The range of hydrolysis rates is inferred from the solution of Eq. (6) and the known experimental range of uptake coefficients.

### Implications for the reactive uptake mechanism

Apart from quantifying a likely range of experimental hydrolysis rates, the analysis of the reaction–diffusion model provides insight into the likely mechanism of reactive uptake. Specifically, the range of uptake values observed upon changing the interfacial hydrolysis rate from 0 to *k*_h_ illustrates that, while interfacial reactivity contributes to the reactive uptake coefficient, it accounts for at most 20%. The interfacial contribution is lower than recent estimates^13^, due to the increased solubility predicted by the MB-nrg model and corresponding higher accommodation coefficient relative to the previous neural network model study. Nevertheless, a significant adsorption-free energy reduces diffusion into the bulk liquid, resulting in an effective renormalized reaction–diffusion length. Absent barriers to solvation, the reaction–diffusion length, ${\ell }_{r}=\sqrt{D/{k}_{{{{{{{{\rm{h}}}}}}}}}}$, would be around 15 nm. However, the barrier to solvation and corresponding free energy minima at the interface results in a propagation length of N_2_O_5_ into the bulk fluid of only around 2 nm (see Fig. 3a). This implies that reactive uptake is affected by interfacial characteristics, even though most of the reaction is predicted to take place in the bulk. It also predicts a very weak aerosol particle size dependence to reactive uptake consistent with some experimental observations^62^.

## Discussion

We report extensive molecular dynamics simulations with many-body potentials of coupled cluster accuracy that quantify the thermodynamics and kinetics of adsorption and solvation of N_2_O_5_ by aqeuous aerosol. The hydrolysis rate of N_2_O_5_ is determined by numerically solving a molecularly detailed reaction–diffusion equation that incorporates these parameters to yield results consistent with the experimentally observed reactive uptake coefficient. This provides a complete quantitative picture of the reactive uptake of N_2_O_5_ by aqueous aerosol. Although most of the hydrolysis is predicted to take place in bulk water, our results highlight the importance of interfacial features at the liquid/vapor interface leading to a relatively short reaction–diffusion length and thus only very weak aerosol particle size dependence.

The framework and parameters determined here can be used as a new starting point for further modeling efforts to predict the reactive uptake of N_2_O_5_ in more complex solutions. It is well known that the reactive uptake can be modulated in the presence of inorganic salts, as in the case of excess nitrate ions^18^. Further, the branching ratios to alternative less soluble products like to ClNO_2_ in the presence of NaCl have been well studied^11,66^. By quantifying the changes to both the thermodynamic and kinetic properties of N_2_O_5_ in the presence of these alternative solutions, advanced molecular models such as the ones used in this work in combination with similar analysis of generalized reaction–diffusion equations incorporating alternative loss mechanics, can be exploited to provide a complete picture of reactive uptake of N_2_O_5_ with the full complexity of field measurements.

## METHODS

### Molecular dynamics simulations

To simulate the uptake of N_2_O_5_ in water we employed the MB-nrg model of N_2_O_5_ in MB-pol water with explicit one-body, two-body, and three-body short-range interactions^16^. The simulation system illustrated in Fig. 1a is made up of 533 water molecules forming a liquid slab measuring 2.416 nm × 2.416 nm in cross-sectional area and 2.772 nm in length. For this slab size, finite size corrections to thermodynamic properties are expected to be minimal^51^. The system is embedded in a simulation domain of the same cross-section and a length of 20 nm in order to accommodate periodic boundary conditions. Simulations were executed with Amber 2020^67^ interfaced to the MBX^68^ library. Ewald summation was employed to describe long-range electrostatics and dispersion interactions using a real-space cutoff of 1.2 nm. Thermodynamic averages were computed within an ensemble of fixed *N* particles, *V* volume, and *T* = 300 K temperature, using a Langevin thermostat and a timestep of 0.5 fs. Kinetic properties were evaluated within a constant energy ensemble with fixed *N* and *V*.

### Umbrella sampling

We computed the free energy to move a gaseous N_2_O_5_ into the liquid water slab using umbrella sampling applied to the center of mass distance along the *z* direction between the water slab and N_2_O_5_^69^. We employed harmonic biasing potentials of the form ${{\Delta }}U(z)=k/2{(z-{z}^{* })}^{2}$ with a spring constant *k* of 2.5 kcal mol^−1^ A^−2^ and 52 independent windows with minima z^*^ spaced evenly between –1.36 and 1.19 nm. Three separate sets of calculations were run, each consisting of 1 ns equilibration time followed by 2.5 ns production time to compute partial histograms. The individual histograms from each window were combined using umbrella integration^70^. Error bars of the free energy profile were computed from the standard deviation for the three independent calculations.

### Diffusion coefficients

Diffusion coefficients were computed from simulations of N_2_O_5_ in bulk liquid water. Details of the calculation of diffusion coefficients from mean-squared displacements in the molecular dynamics simulations are discussed in the Supplementary Methods.

## Supplementary information

Supplementary Information

Peer Review File

## Data Availability

Data supporting the findings of this study are included in the article and a Source Data file is provided with this paper. In addition, the data related to this publication including simulation input files can be accessed from the NSF-CAICE Data Repository^71^ (10.6075/J0FF3SHB). Source data are provided with this paper.

## Source data

Source Data

## Code availability

This study utilized Amber 20 interfaced to MBX (http:// paesanigroup.ucsd.edu/software/mbx.html). The software for solving the reaction–diffusion model is included in the Supplementary Material and can be accessed from the NSF-CAICE Data Repository^71^ (10.6075/J0FF3SHB).
