# Supplementary material for: Uptake of N2O5 by aqueous aerosol unveiled using chemically accurate many-body potentials
Source: Nat Commun. 2022 Mar 10;13:1266. doi: 10.1038/s41467-022-28697-8 (PMC8913772; doi:10.1038/s41467-022-28697-8)
Supplement: Supplementary file 1 — Supplementary Information [file 41467_2022_28697_MOESM1_ESM.pdf]

Supplementary Information

Uptake of N_2O_5 by aqueous aerosol unveiled using chemically accurate many-body potentials

Vinícius Wilian D. Cruzeiro, Mirza Galib, David T. Limmer, and Andreas W. Götz

SUPPLEMENTARY NOTES

Summary of Thermodynamic and Kinetic Parameters

Supplementary Figure 1 shows the free energy profile of N_2O_5 solvation in water along with characteristic snapshots of N_2O_5 in bulk water, at the liquid/vapor interface, and in vapor. The adsorption free energy for N_2O_5 adsorbing from the vapor to the water liquid/vapor interface is labeled as ΔF_{a}, the solvation free energy of gaseous N_2O_5 as ΔF_{s}, and the free energy barrier for desolvation of N_2O_5 from bulk water to the interface is ΔF_{b}. Consequently, the free energy of solvation for surface adsorbed N_2O_5 is given as $\Delta F_{\mathrm{s}} - \Delta F_{\mathrm{a}}$, and the barrier to solvation is $\Delta F_{\mathrm{s}} - \Delta F_{\mathrm{a}} + \Delta F_{\mathrm{b}}$. Supplementary Figure 1 also defines the rate constants for adsorption and evaporation (k_{a}, k_{e}) and solvation and desolvation (k_{s}, k_{d}).

Supplementary Table 1 collects all thermodynamic and kinetic parameters for N_2O_5 uptake to water that have been determined in this work as defined in Supplementary Figure 1. We report free energies in kcal/mol to complement the presentation in the main text.

SUPPLEMENTARY METHODS

Calculation of Diffusion Coefficients

Bulk diffusion coefficients from mean squared displacements

The bulk diffusion constant, D, was evaluated by computing the mean-squared displacement of an N_2O_5 molecule over 100 trajectories. The initial coordinates and velocities for each trajectory were taken from uncorrelated structures of a simulation performed in the canonical ensemble with a target temperature of 300 K and a volume that had been previously equilibrated in a simulation at ambient temperature and pressure. Each of the 100 trajectories then evolved in the microcanonical ensemble for 100 ps. For each trajectory, multiple predictions for the mean-squared displacement were obtained by taking different slices 10 ps long of the trajectory. All trajectories were split into four groups of 25, and one value of D was computed for each one. The value of D and its corresponding error reported in the main text are taken from the average and standard deviation of the D predictions across the four groups.

Following the same procedure, we have also computed the bulk diffusion constant for water using information from all the 272 water molecules in the simulation box. We obtained a value of $(2.34 \pm 0.02) \times 10^{-5}$ cm^2/s, which is in close agreement with the value previously reported for MB-pol at the same temperature for a simulation box of similar size.[1]

In order to infer the effect of sampling, we evaluated the bulk diffusion constant of water using data for a single water molecule that has been selected randomly. A value of $(2.25 \pm 0.08) \times 10^{-5}$ cm^2/s is obtained. This value agrees within error bars with the prediction using data from all water molecules.

Supplementary Figure 2 shows the average and standard deviation of the mean-squared displacement across all 100 trajectories. As one would expect, the standard deviation when using data for all molecules is significantly smaller than when considering a single water molecule. On the other hand, the standard deviation obtained for a single water molecule is comparable to the one for N_2O_5, since these predictions contain the same amount of sampling.

Diffusion in the interfacial region

To compute the z dependence of the diffusion constant, $D(z)$, we follow a procedure due to Hummer, valid for simulations where the reaction coordinate z is restrained to a particular window with a harmonic bias potential.[2] Specifically, an autocorrelation function of $z(t)$ is computed under a harmonic bias. Its value at $t = 0$ and decay time can be used to compute $D(z)$, envisioning that the dynamics is well described by an Ornstein-Uhlenbeck process. Both values are obtained from the constrained simulations using the biasing potentials employed in the umbrella sampling calculations. The profile is largely featureless, plateauing for large and small z. The interfacial diffusivity quoted in the main text is evaluated at the Gibbs dividing surface.

Assessment of sampling quality in the umbrella sampling simulations

To evaluate the quality of sampling in the three independent umbrella sampling simulations that we performed, in Supplementary Figure 3 we present histograms of the reaction coordinate z for each window in the umbrella sampling simulations. As can be observed in the figure, each window exhibits good overlap with the neighboring windows, with a significant overlap extending up to the third neighbor. This observation is true for all independent umbrella sampling simulations. Therefore, we confirm excellent sampling along all relevant values of the z coordinate.

SUPPLEMENTARY TABLES

Supplementary Table 1: Free energies and kinetic properties

Free Energy of Adsorption	ΔF_{a}	(-3.69 ± 0.06) kcal/mol
Free Energy of Solvation	ΔF_{s}	(-2.56 ± 0.06) kcal/mol
Free Energy Barrier to Desolvation	ΔF_{b}	(0.48 ± 0.06) kcal/mol
Rate of Adsorption	k_{a}	57 nm/ns
Rate of Evaporation	k_{e}	0.11 nm/ns
Rate of Solvation	k_{s}	51 ns^{-1}
Rate of Desolvation	k_{d}	340 ns^{-1}
Hydrolysis Rate[a]	k_{h}	$(4 \pm 3) \times 10^{-2}$ ns^{-1}

SUPPLEMENTARY FIGURES

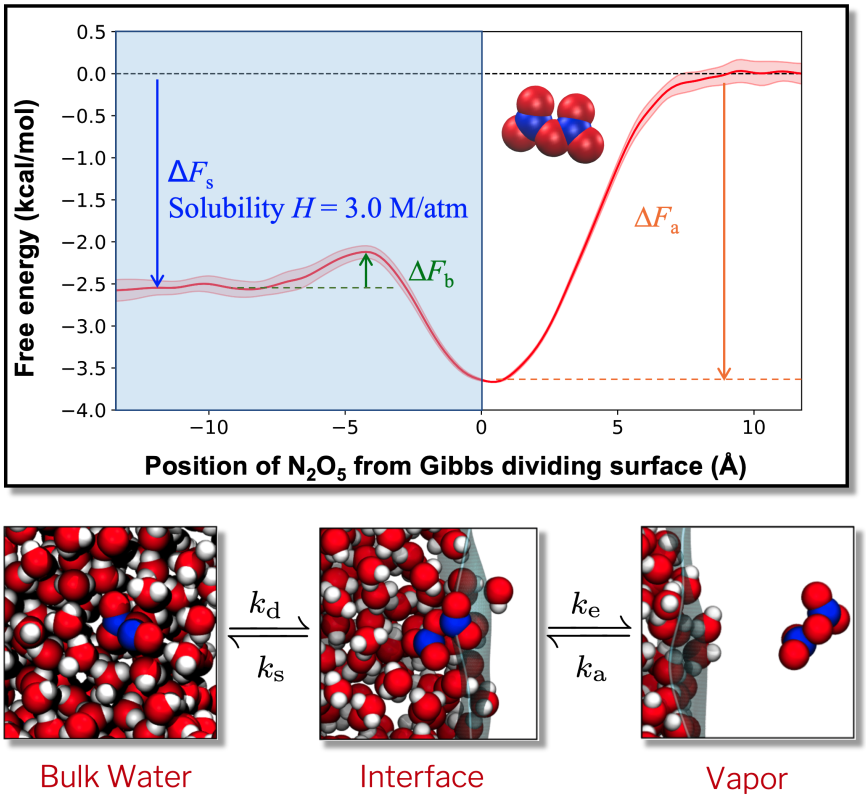

Supplementary Figure 1: Thermodynamics and kinetics of N_2O_5 adsorption and solvation. Free energy profile with definitions of adsorption free energy ΔF_a, solvation free energy ΔF_s, and free energy barrier for desolvation ΔF_b (top) along with characteristic snapshots of N_2O_5 (bottom). The shaded region in the free energy profile corresponds to one standard deviation error bars. Adsorption and evaporation take N_2O_5 between the vapor and liquid/vapor interface with rate constants k_a and k_e. Solvation and desolvation takes N_2O_5 between the liquid/vapor interface and bulk water with rate constants k_s and k_d.

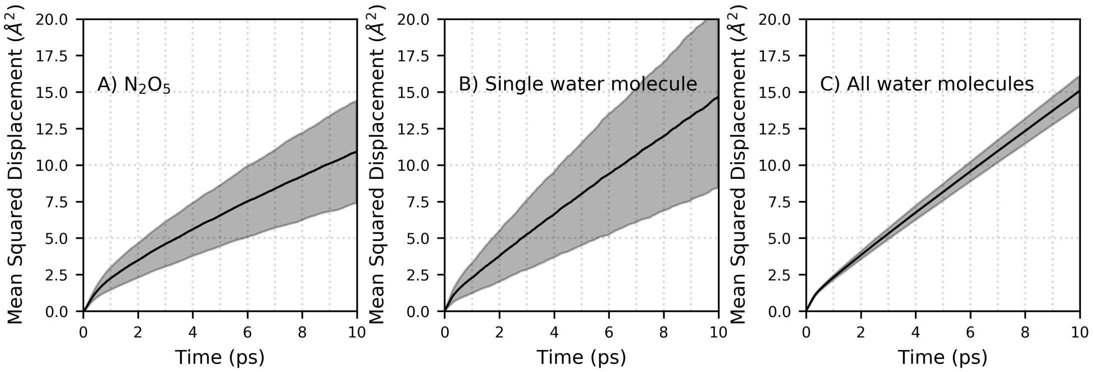

Supplementary Figure 2: Mean squared displacement in bulk for A) the N_2O_5 molecule, B) a single water molecule randomly selected, and C) all water molecules. The shaded region corresponds to one standard deviation error bars.

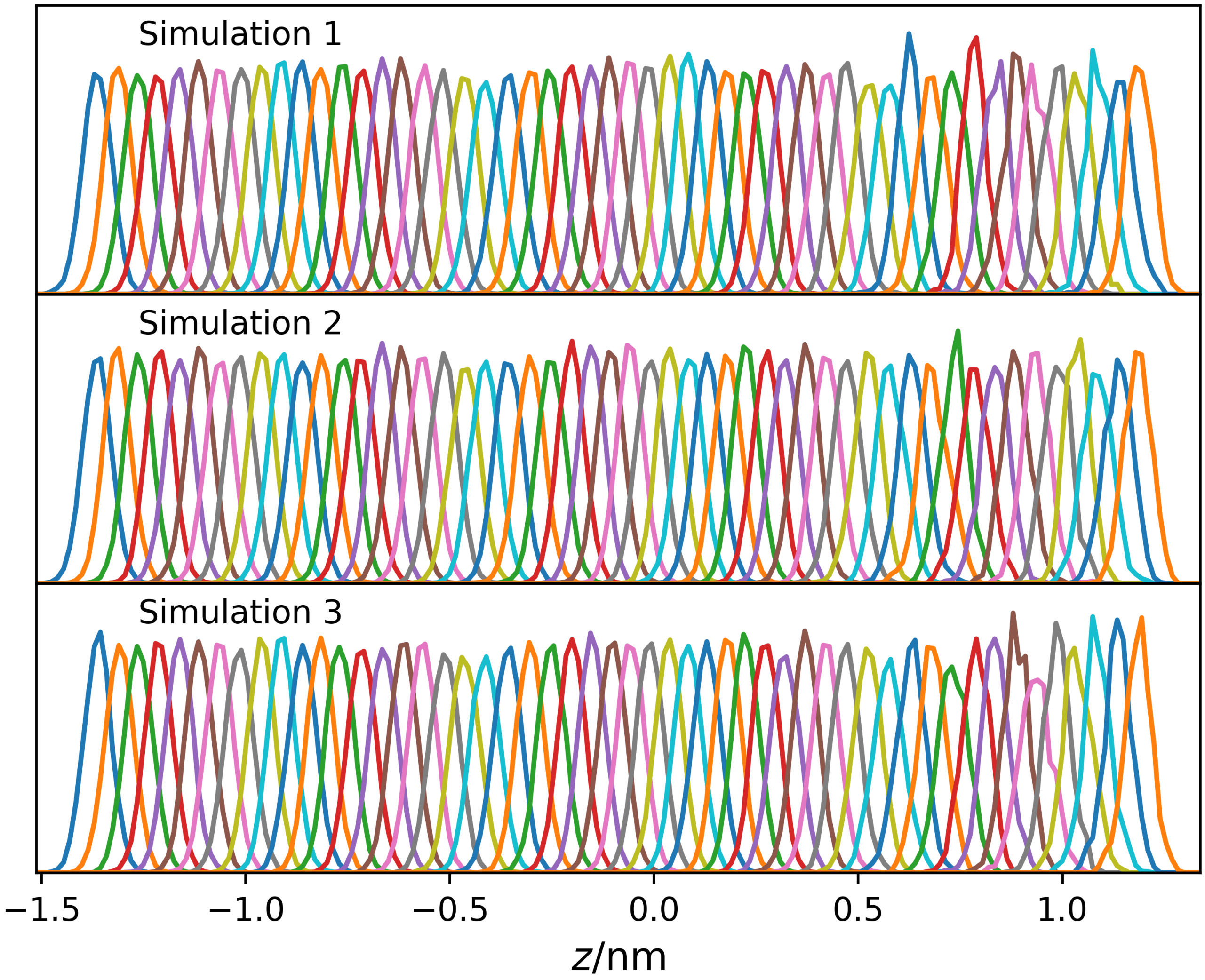

Supplementary Figure 3: Distribution of values for the reaction coordinate z for each window for each independent umbrella sampling simulation.

SUPPLEMENTARY REFERENCES

---

[1] Reddy, S. K. *et al.* On the accuracy of the MB-pol many-body potential for water: Interaction energies, vibrational frequencies, and classical thermodynamic and dynamical properties from clusters to liquid water and ice. *The Journal of Chemical Physics* **145**, 194504 (2016).

[2] Hummer, G. Position-dependent diffusion coefficients and free energies from Bayesian analysis of equilibrium and replica molecular dynamics simulations. *New Journal of Physics* **7**, 34 (2005).
