# Supplementary figures and images for: Uptake of N2O5 by aqueous aerosol unveiled using chemically accurate many-body potentials
Source: Nat Commun. 2022 Mar 10;13:1266. doi: 10.1038/s41467-022-28697-8 (PMC8913772; doi:10.1038/s41467-022-28697-8)

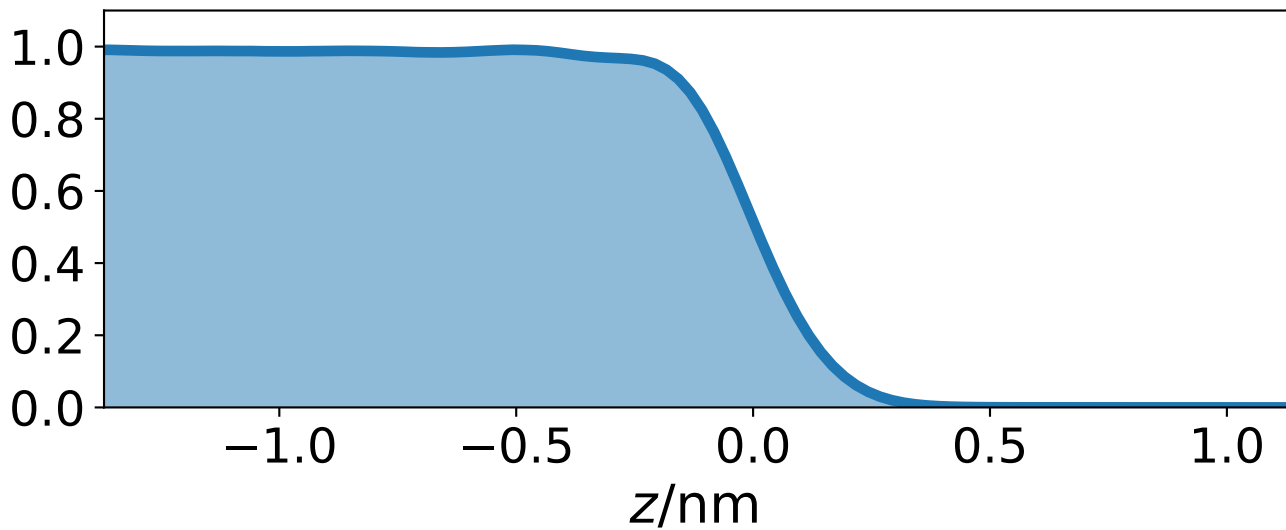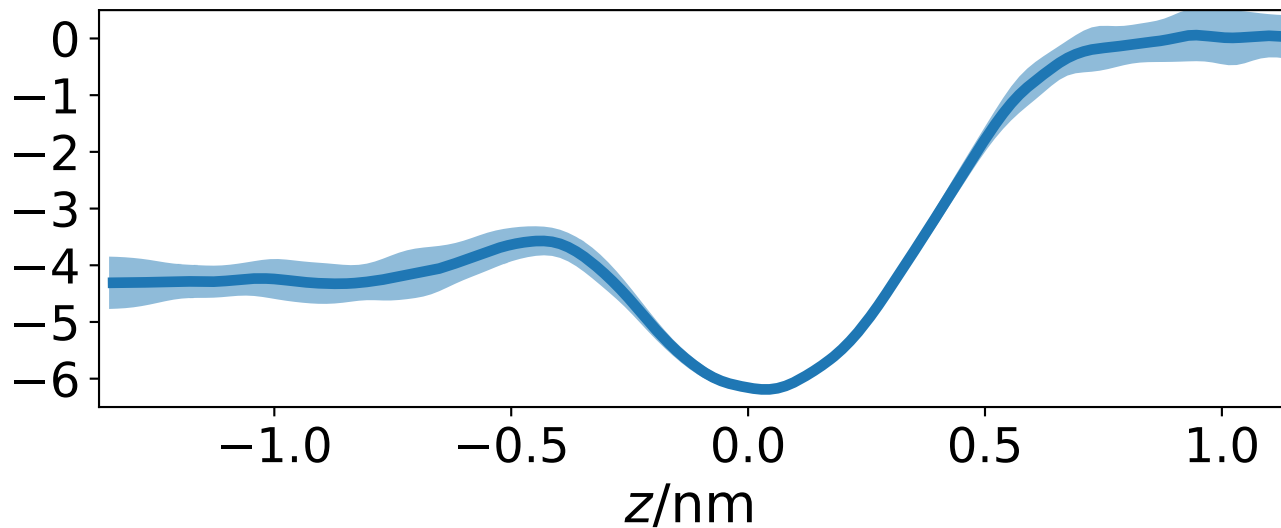

Supplement: Supplementary file 3 — Source Data [file 41467_2022_28697_MOESM3_ESM.zip › n2o5-uptake-data/Fig1/Fig1.pdf]

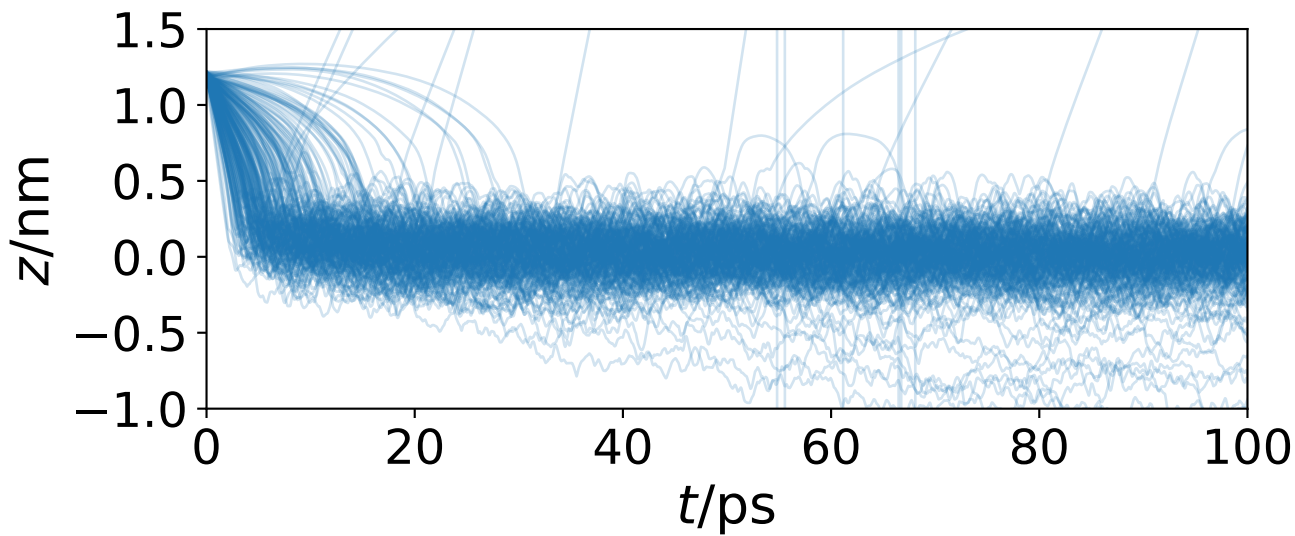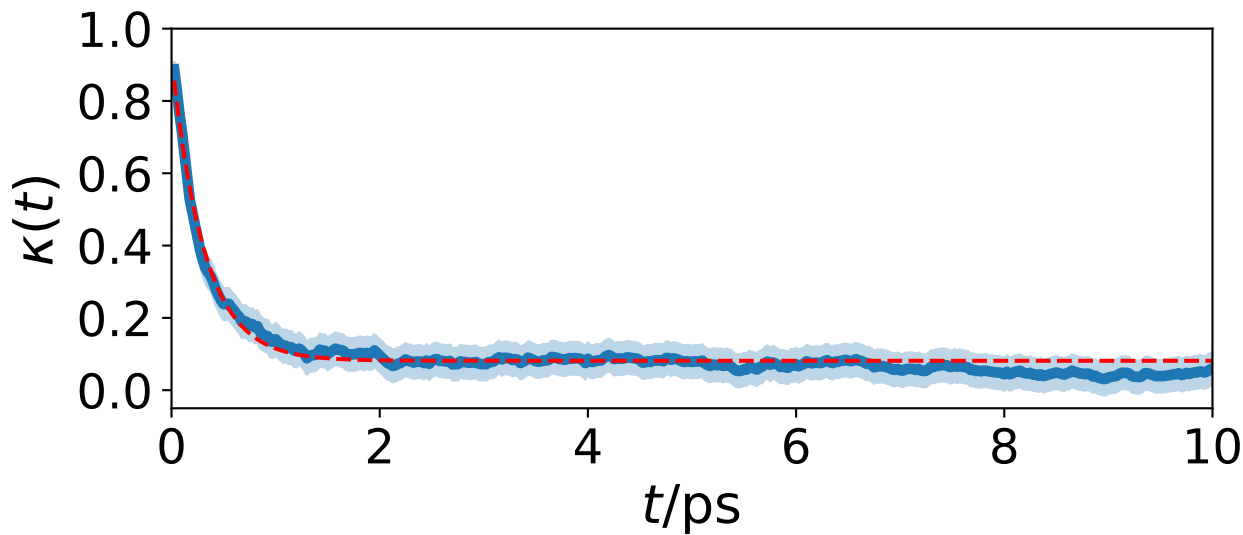

Supplement: Supplementary file 3 — Source Data [file 41467_2022_28697_MOESM3_ESM.zip › n2o5-uptake-data/Fig2/Fig2.pdf]
