# Supplementary material for: Uptake of N2O5 by aqueous aerosol unveiled using chemically accurate many-body potentials
Source: Nat Commun. 2022 Mar 10;13:1266. doi: 10.1038/s41467-022-28697-8 (PMC8913772; doi:10.1038/s41467-022-28697-8)
Supplement: Supplementary file 3 — Source Data [file 41467_2022_28697_MOESM3_ESM.zip › n2o5-uptake-data/Fig3/Fig3.pdf]

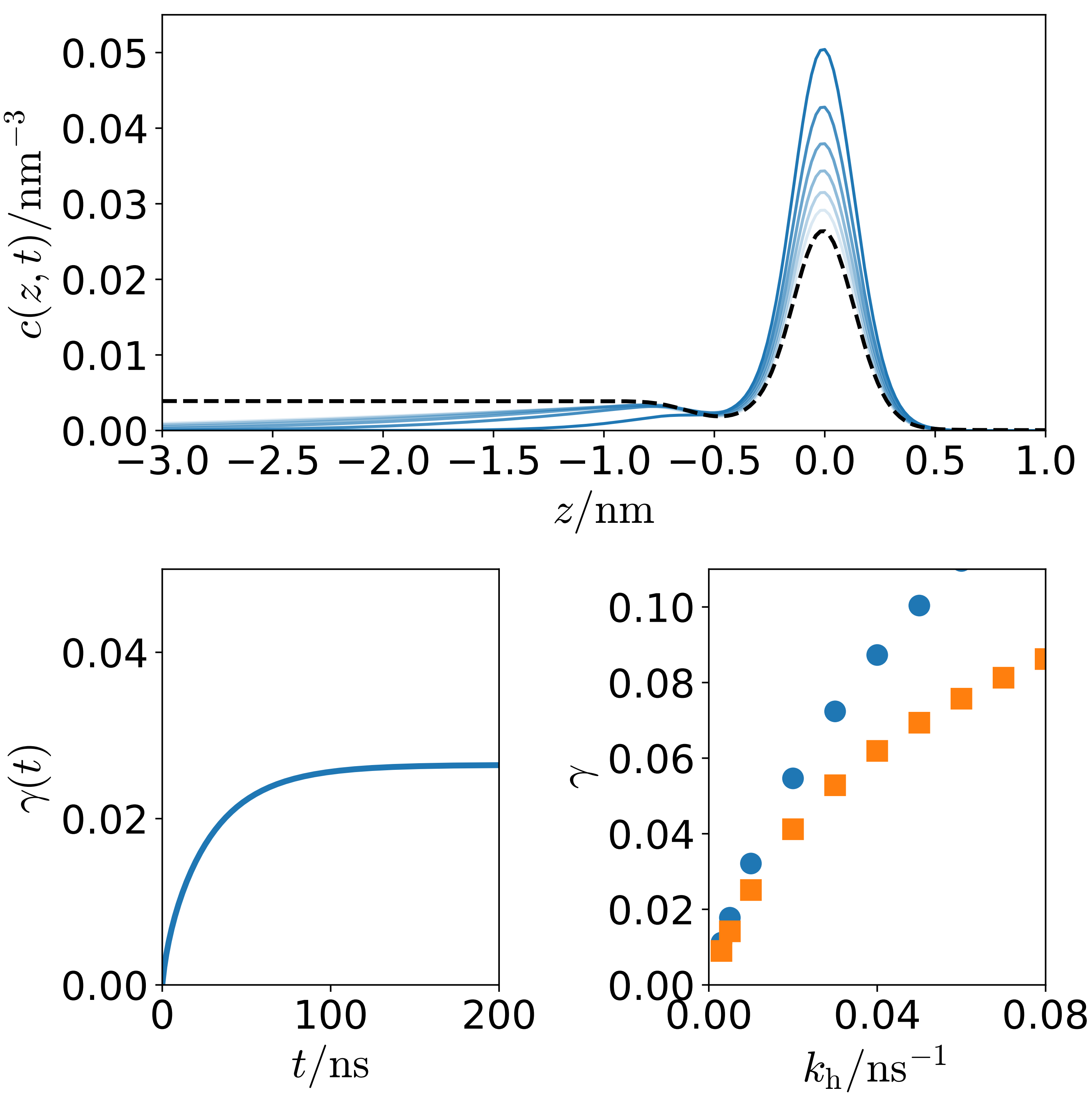

$c(z,t)/\mathrm{nm}^{-3}$
0.05
0.04
0.03
0.02
0.01
0.00
−3.0 −2.5 −2.0 −1.5 −1.0 −0.5 0.0 0.5 1.0
$z/\mathrm{nm}$
$\gamma(t)$
0.04
0.02
0.00
0 100 200
$t/\mathrm{ns}$
$\gamma$
0.10
0.08
0.06
0.04
0.02
0.00
0.00 0.04 0.08
$k_\mathrm{h}/\mathrm{ns}^{-1}$
