# Supplementary material for: Uptake of N2O5 by aqueous aerosol unveiled using chemically accurate many-body potentials
Source: Nat Commun. 2022 Mar 10;13:1266. doi: 10.1038/s41467-022-28697-8 (PMC8913772; doi:10.1038/s41467-022-28697-8)
Supplement: Supplementary file 2 — Peer Review File [file 41467_2022_28697_MOESM2_ESM.pdf]

nature portfolio

Peer Review File

Uptake of N2O5 by aqueous aerosol unveiled using chemically accurate many-body potentials

Open Access This file is licensed under a Creative Commons Attribution 4.0 International License, which permits use, sharing, adaptation, distribution and reproduction in any medium or format, as long as you give appropriate credit to the original author(s) and the source, provide a link to the Creative Commons license, and indicate if changes were made. In the cases where the authors are anonymous, such as is the case for the reports of anonymous peer reviewers, author attribution should be to 'Anonymous Referee' followed by a clear attribution to the source work. The images or other third party material in this file are included in the article's Creative Commons license, unless indicated otherwise in a credit line to the material. If material is not included in the article's Creative Commons license and your intended use is not permitted by statutory regulation or exceeds the permitted use, you will need to obtain permission directly from the copyright holder. To view a copy of this license, visit http://creativecommons.org/licenses/by/4.0/.

REVIEWER COMMENTS

Reviewer #1 (Remarks to the Author):

The authors present a simulation study of the reactive uptake of N2O5 into aqueous aerosol droplets. Using a newly developed many-body model for N2O5 and water based on coupled cluster theory calculations, they performed simulations of a single N2O5 molecule in the vicinity of water's liquid-vapor interface. Using umbrella sampling simulations they obtained the free energy profile of solvating an N2O5 molecule, which revealed a global minimum in free energy when the N2O5 molecules is adsorbed to the liquid-vapor interface, as well as a free energy barrier to incorporation into the bulk liquid. The authors then used this free energy profile, along with key rate constants obtained from the simulation results, within a reaction-diffusion equation to model the reactive uptake of N2O5 in the presence of hydrolysis. Importantly, the authors show, with a state-of-the-art many-body computational model, the importance of considering interfacial adsorption in understanding the reactive uptake of N2O5 and in interpreting available experimental data.

The study is carefully done and clearly presented. The strategy of coupling the simulation results with a reaction diffusion theoretical framework creatively enables insight into the reactive system despite the simulations' lack of ability to model reactive events. The authors place their results in context of prior simulations and experimental results, and highlight key gaps in the interpretation of available experimental data. The fundamental insight of how interfacial adsorption controls the uptake of N2O5 is impactful, and the approach and methods used should serve as an inspiration for those tackling similar systems. I feel that the conclusions are sound and well-supported by the data, and the manuscript is clearly written. I only have a few relatively minor clarification comments and suggestions:

1) The authors motivate the study by discussing uptake of N2O5 in tropospheric aerosols. The current simulations are performed at ambient conditions (T = 300 K), but temperatures range wildly in the troposphere, extending down to ~200 K. How robust/transferrable are these results if the temperature were to decrease? Are all of the experimental results the authors discuss also performed under ambient conditions?

2) When comparing to experiments and prior simulations, qualitative language is often used, e.g., 'in line with' on line 154, and 'faster'/'slower' on lines 330-332. When possible, can the authors report the specific values reported in those previous studies to facilitate easier comparison with the present results?

3) Can the authors provide the histograms of the z coordinate sampled in the umbrella sampling simulations in the supporting information so that readers can evaluate the overlap of windows/quality of sampling?

4) On line 296, the use of the phrase 'in the absence of irreversible reactions' implies that there could still be reversible reactions present in the system. However, based on my reading, it seems to me that the authors instead mean 'in the absence of any reactions', given that the equilibrium free energy profile was obtained with a non-reactive model. Can the authors clarify?

5) The authors provide a rather long list of papers (Refs. 26-43) to demonstrate MB-pol's accuracy in reproducing the properties of water, though many of those papers are not directly relevant to the air/water interfacial system considered in this work (i.e., those focusing on crystalline ice). However, there is a recent paper on MB-pol's ability to reproduce water's vapor-liquid equilibrium and interfacial properties, which helps bolster the argument that MB-nrg models perform well in such interfacial geometries [Muniz et al., J. Chem. Phys. 154, 211103 (2021)]. The authors may wish to consider refocusing the reference list to those papers most relevant to the system at hand, and/or consider including this work as well.

Reviewer #2 (Remarks to the Author):

In this manuscript, Cruzeiro and coworkers investigate the reactive and non-reactive uptake of N2O5 in water an important process for atmospheric chemistry. To this scope, the authors perform molecular dynamics simulations using MB-nrg, an accurate forcefield that is an extension of the popular MB-pol model to treat this system.

It is important to point out that Galib and Limmer addressed reactive adsorption of N2O5 at water surfaces in a recent work published in Science (Ref. 13), where they used a different type of interaction potential based on neural networks. This work corrects some of the numerical results presented in Ref.13, bringing the theoretical predictions in closer agreement to experiments. The thermodynamics of non-reactive uptake is here studied by means of a free-energy calculation that shows a minimum at the interface, and the kinetics is investigated by a scattering numerical experiment.

While the forcefield used here is more accurate than that in Ref.13, it is not "reactive", i.e. it does not allow one to simulate directly the hydrolysis of N2O5. To overcome this issue reactive uptake is modeled with a (continuum) Smoluchowski equation that incorporates the results of the non-reactive molecular simulations and allows the authors to determine a range for the hydrolysis rate, as well as the penetration depth of N2O5 in water.

This work highlights once more the importance of the water surface in this process, and it provides predictions that should be more reliable than the previous ones as for the parameters that control the reactive uptake of N2O5 in water droplets.

The paper is well-written: very clear and enjoyable. The presentation of the results is insightful and logical. The methods section (and SI) provides sufficient technical details to reproduce the simulations, including input files and source codes.

For these reasons, I would be glad to see this work published, but I would ask the authors to highlight the novelty of this work with respect to their former paper (Ref.13) before fully endorsing its publication in Nature Comm.

Additionally, I would like to see the following issues addressed before publication:

1- it is often mentioned that the quantities calculated by molecular simulations are in more or less good agreement with experiments: while references are provided to the experimental works, it would be useful for the readers that the experimental values are reported explicitly in the text.

2- The calculations of the adsorption and evaporation rates are not particularly enlightening, as they confirm what one would infer from the free energy calculations at equilibrium. As the authors mention that the accommodation coefficient S=0.96 is a lower bound, what is the uncertainty of S? How sensitive are their results to this parameter? Would it make a difference to use S=1?

3- I suspect that the width of the interface of 0.06 nm reported in line 255 is a typo. The interfacial region should be one order of magnitude thicker.

Reviewer #3 (Remarks to the Author):

Review NCOMMS-21-37451-T

This work reports an extensive MD investigation of relevant interphase and reactive phenomena related to the adsorption and reactivity of an important atmospheric species like N_2O_5 in water. The research used various MD and free energy methods taking advantage of a recently derived ML potential for N_2O_5 in aqueous environments at coupled cluster level, which is indeed remarkable.

The article is well-written, every detail and possible pitfalls of the results and conclusion is supported by a satisfactory selection of relevant literature, both theoretical and experimental, and by very convincing arguments and deep computational investigations.

The methodology seems sound and the results look transparent and clear.

To be honest, I am not completely persuaded that this work will be particularly significant to the field of molecular simulations or to the one of atmospheric chemistry. Nonetheless, it represents

indeed a very solid and clear piece of research that puts together many state-of-the-art techniques and, thus, I prefer to hand this judgment to the editor only.

Here below I report a couple of minor comments:

1) P. 2 L. 133 & Fig. 1: the authors report the free energy surface of the adsorption/solvation step of N2O5 in H2O. The correctly report that, I quote "the free energy is non-monotonic and exhibits a global minimum approximately centered at the Gibb's dividing surface". In my opinion, the minimum must coincide with the dividing surface and here it is slightly shifted toward positive values. This may be the consequence of non-equilibrium conditions. Please provide numerical evidence that this is not the case.

2) P. 3. Concerning the calculation of Henry's constant and other thermodynamics parameters, I wonder whether these results may be strongly affected by finite size problems. Please comment on that and provide evidence that your simulation box and conditions are accounting for FS effects. In the supp. Material (P. 4) it is reported that the diffusion coefficients is similar to a previous calculation with similar box size. Maybe a shorter simulation of some other parameter relevant to this work with a larger box may support your choice.

UNIVERSITY OF CALIFORNIA, SAN DIEGO UCSD

BERKELEY • DAVIS • IRVINE • LOS ANGELES • MERCED • RIVERSIDE • SAN DIEGO • SAN FRANCISCO SANTA BARBARA • SANTA CRUZ

DR. ANDREAS W. GOETZ
SAN DIEGO SUPERCOMPUTER CENTER
9500 GILMAN DR, MC0505
LA JOLLA, CA 92093–0505

PHONE: (858) 822–4771
FAX: (858) 534–5117
EMAIL: AGOETZ@SDSC.EDU

December 12, 2021

RE: Manuscript **NCOMMS-21-37451-T**

"Uptake of N_2O_5 by aqueous aerosol unveiled using chemically accurate many-body potentials"
Vinícius W. D. Cruzeiro, M. Galib, David T. Limmer, Andreas W. Götz

Point-by-point response to reviewers' comments.

Reviewer #1 (Remarks to the Author):

The authors present a simulation study of the reactive uptake of N2O5 into aqueous aerosol droplets. Using a newly developed many-body model for N2O5 and water based on coupled cluster theory calculations, they performed simulations of a single N2O5 molecule in the vicinity of water's liquid-vapor interface. Using umbrella sampling simulations they obtained the free energy profile of solvating an N2O5 molecule, which revealed a global minimum in free energy when the N2O5 molecules is adsorbed to the liquid-vapor interface, as well as a free energy barrier to incorporation into the bulk liquid. The authors then used this free energy profile, along with key rate constants obtained from the simulation results, within a reaction-diffusion equation to model the reactive uptake of N2O5 in the presence of hydrolysis. Importantly, the authors show, with a state-of-the-art many-body computational model, the importance of considering interfacial adsorption in understanding the reactive uptake of N2O5 and in interpreting available experimental data.

Comment 0
The study is carefully done and clearly presented. The strategy of coupling the simulation results with a reaction diffusion theoretical framework creatively enables insight into the reactive system despite the simulations' lack of ability to model reactive events. The authors place their results in context of prior simulations and experimental results, and highlight key gaps in the interpretation of available experimental data. The fundamental insight of how interfacial adsorption controls the uptake of N2O5 is impactful, and the approach and methods used should serve as an inspiration for those tackling similar systems. I feel that the conclusions are sound and well-supported by the data, and the manuscript is clearly written. I only have a few relatively minor clarification comments and suggestions:

Response 0
We thank the reviewer for their kind comments and believe that in addressing their requests for clarification below, that our manuscript is clearer and more accurate.

Comment 1

1) The authors motivate the study by discussing uptake of N2O5 in tropospheric aerosols. The current simulations are performed at ambient conditions (T = 300 K), but temperatures range wildly in the troposphere, extending down to 200 K. How robust/transferrable are these results if the temperature were to decrease? Are all of the experimental results the authors discuss also performed under ambient conditions?

Response 1

We thank the reviewer for raising this interesting point and agree that atmospherically relevant conditions for aerosol chemistry extend well beyond ambient conditions. The temperature dependence of N_2O_5 reactive uptake has been studied experimentally, and has been found to be very weakly negative changing at most by a factor of 50% down to 200 K [1,2]. Indeed estimates of the uptake coefficient on liquid water and ice are routinely reported to be quantitatively similar [2]. Because of this weak temperature dependence, we expect our results are very transferable and we have not explored other thermodynamic conditions. The experimental values compared to in the main text have all been evaluated at 300 K.

[1] J. M. Van Doren et al., *J. Phys. Chem.* **94**, 3265–3269 (1990).
[2] C. George et al., *J. Phys. Chem. C* **98**, 8780–8784 (1994).

Comment 2

2) When comparing to experiments and prior simulations, qualitative language is often used, e.g., 'in line with' on line 154, and 'faster'/'slower' on lines 330-332. When possible, can the authors report the specific values reported in those previous studies to facilitate easier comparison with the present results?

Response 2

We agree with the reviewer that more quantitative language should be used. Concerning line 154 (line 158 in revised manuscript) we have added "*value is in line with typical inferences from experiment which range between 1 to 10 M/atm, though its direct measurement is hindered by the facile hydrolysis of* N_2O_5." Concerning lines 330-332 (lines 339-342 in revised manuscript), we have added "*These rates are slower than those computed directly from a previous neural network model (0.2 ns^{-1}),[13] but faster than inferred experimentally (0.002 ns^{-1}).[64]*"

Comment 3

3) Can the authors provide the histograms of the z coordinate sampled in the umbrella sampling simulations in the supporting information so that readers can evaluate the overlap of windows/quality of sampling?

Response 3

We have added a figure to the SI consistent with the request of the reviewer, along with a short discussion. The histogram confirms good overlap of the umbrella windows and hence excellent sampling along the collective variable (z-coordinate).

Comment 4

4) On line 296, the use of the phrase 'in the absence of irreversible reactions' implies that there could still be reversible reactions present in the system. However, based on my reading, it seems to me that the authors instead mean 'in the absence of any reactions', given that the equilibrium free energy profile was obtained with a non-reactive model. Can the authors clarify?

Response 4

The reviewer is correct that this was a misstatement. Indeed, we mean in the absence of any reactions, as any not just those that are irreversible, would affect the steady-state concentration profile of N_2O_5. We have

corrected the main text to read: "*In the absence of any reactions,*"

Comment 5
5) The authors provide a rather long list of papers (Refs. 26-43) to demonstrate MB-pol's accuracy in reproducing the properties of water, though many of those papers are not directly relevant to the air/water interfacial system considered in this work (i.e., those focusing on crystalline ice). However, there is a recent paper on MB-pol's ability to reproduce water's vapor-liquid equilibrium and interfacial properties, which helps bolster the argument that MB-nrg models perform well in such interfacial geometries [Muniz et al., J. Chem. Phys. 154, 211103 (2021)]. The authors may wish to consider refocusing the reference list to those papers most relevant to the system at hand, and/or consider including this work as well.

Response 5
In consideration to the reviewer's suggestion, we have added and emphasized the referred reference. We thank the reviewer for pointing this out.

Reviewer #2 (Remarks to the Author):

In this manuscript, Cruzeiro and coworkers investigate the reactive and non-reactive uptake of N2O5 in water an important process for atmospheric chemistry. To this scope, the authors perform molecular dynamics simulations using MB-nrg, an accurate forcefield that is an extension of the popular MB-pol model to treat this system.

It is important to point out that Galib and Limmer addressed reactive adsorption of N2O5 at water surfaces in a recent work published in Science (Ref. 13), where they used a different type of interaction potential based on neural networks. This work corrects some of the numerical results presented in Ref.13, bringing the theoretical predictions in closer agreement to experiments. The thermodynamics of non-reactive uptake is here studied by means of a free-energy calculation that shows a minimum at the interface, and the kinetics is investigated by a scattering numerical experiment.

While the forcefield used here is more accurate than that in Ref.13, it is not "reactive", i.e. it does not allow one to simulate directly the hydrolysis of N2O5. To overcome this issue reactive uptake is modeled with a (continuum) Smoluchowski equation that incorporates the results of the non-reactive molecular simulations and allows the authors to determine a range for the hydrolysis rate, as well as the penetration depth of N2O5 in water.

This work highlights once more the importance of the water surface in this process, and it provides predictions that should be more reliable than the previous ones as for the parameters that control the reactive uptake of N2O5 in water droplets.

The paper is well-written: very clear and enjoyable. The presentation of the results is insightful and logical. The methods section (and SI) provides sufficient technical details to reproduce the simulations, including input files and source codes.

Comment 0
For these reasons, I would be glad to see this work published, but I would ask the authors to highlight the novelty of this work with respect to their former paper (Ref.13) before fully endorsing its publication in

Nature Comm.

Response 0

We thank the reviewer for their positive assessment of our work, and for providing us an opportunity to revise our manuscript to highlight its novelty. As the reviewer correctly states, this present manuscript follows up an earlier study by Galib and Limmer. That study suggested that the long standing perspective of the reactive uptake of N_2O_5 being determined by bulk processes like solvation and bulk hydrolysis may be incorrect, and rather uptake could be determined by interface processes of adsorption, evaporation and interfacial hydrolysis. However, the inaccuracy of the model employed by Galib and Limmer made it impossible to conclude this conclusively or to provide valuable parameters to guide further experiments. The current manuscript, employing a chemically accurate model potential fills these important gaps. In making quantitatively accurate predictions, it validates the basic picture proposed by Galib and Limmer, bringing it to bear on the real world and provides important thermodynamic parameters that are not possible to measure experimentally like the solvation and adsorption free energies, which will undoubtedly be used by researchers in the field as they apply this new perspective to increasingly more complex systems.

In order to highlight this novelty we have added to line 79, "*This is the aim of the current work, to apply a quantitatively accurate potential to extract the thermodynamic and kinetic properties underpinning the uptake of* N_2O_5 *into water.*"

Comment 1

Additionally, I would like to see the following issues addressed before publication:
1- it is often mentioned that the quantities calculated by molecular simulations are in more or less good agreement with experiments: while references are provided to the experimental works, it would be useful for the readers that the experimental values are reported explicitly in the text.

Response 1

We agree with the reviewer that our statements should be made quantitative when possible. We have added explicit values for the experimentally inferred Henry's law constant and hydrolysis rate as an effort to do this. A difficulty that arises in some of the cases alluded to in the manuscript is that the experimental values either have large uncertainties or have not been measured directly, but rather inferred using models with questionable accuracy. In these latter cases we have left our statements qualitative.

Comment 2

2- The calculations of the adsorption and evaporation rates are not particularly enlightening, as they confirm what one would infer from the free energy calculations at equilibrium. As the authors mention that the accommodation coefficient S=0.96 is a lower bound, what is the uncertainty of S? How sensitive are their results to this parameter? Would it make a difference to use S=1?

Response 2

The reviewer raises a reasonable point. First, the uncertainties of S are ± 0.06 as computed from the standard error of a Poisson process using our 250 independent scattering calculations. More saliently however, the deviation from S=1 is not of significant relevance. Within our model, uptake is proportional to S, so changing it between 0.9 and 1 changes γ proportionally, which is much smaller than the uncertainty in its experimental evaluation. We have clarified this point in the main text by writing on line 216, "$S = 0.96 \pm 0.06$, *relating the probability of being accommodated at the interface upon collision, consistent with previous simulations.[15] This is likely a lower bound as some of the back scattering events can be attributable to equilibration at the*

interface followed by subsequent evaporation. The near unity value implies a lack of a barrier to adsorption, that subsequent evaporation is analogously limited only by the free energy of adsorption, and that uptake is not significantly influenced by this initial thermal accommodation."

Comment 3
3- I suspect that the width of the interface of 0.06 nm reported in line 255 is a typo. The interfacial region should be one order of magnitude thicker.

Response 3
We thank the reviewer for catching this typo. Indeed, we meant to write 0.6 nm. This has been corrected in the text.

Reviewer #3 (Remarks to the Author):

Review NCOMMS-21-37451-T

This work reports an extensive MD investigation of relevant interphase and reactive phenomena related to the adsorption and reactivity of an important atmospheric species like N2O5 in water. The research used various MD and free energy methods taking advantage of a recently derived ML potential for N2O5 in aqueous environments at coupled cluster level, which is indeed remarkable.

The article is well-written, every detail and possible pitfalls of the results and conclusion is supported by a satisfactory selection of relevant literature, both theoretical and experimental, and by very convincing arguments and deep computational investigations.

Comment 0
The methodology seems sound and the results look transparent and clear. To be honest, I am not completely persuaded that this work will be particularly significant to the field of molecular simulations or to the one of atmospheric chemistry. Nonetheless, it represents indeed a very solid and clear piece of research that puts together many state-of-the-art techniques and, thus, I prefer to hand this judgment to the editor only.

Response 0
We thank the reviewer for their comments and for finding our results convincing. We would note that this particular question of the reactive uptake of N_2O_5 is of considerable interest in atmospheric chemistry, due to the fact that this loss mechanism is a significant factor to the overall NOx concentration in the atmosphere. Due to its fast hydrolysis kinetics, experimental studies have been unable to definitely pin down basic thermodynamic and kinetic properties of N_2O_5's solvation and reaction, despite decades of efforts. This is precisely why simulations with chemical accuracy are important, as these can supply parameters to researchers in the field as they extrapolate beyond the pure aqueous aerosol considered here. The simulation framework and parameters determined here enable further modeling efforts to predict reactive uptake not only of N_2O_5 but also other gas molecules and can be exploited to provide a complete and quantitative picture of these processes with the full complexity of field measurements that have previously been difficult to rationalize.

Here below I report a couple of minor comments:

Comment 1

1) P. 2 L. 133 & Fig. 1: the authors report the free energy surface of the adsorption/solvation step of N2O5 in H2O. The correctly report that, I quote "the free energy is non-monotonic and exhibits a global minimum approximately centered at the Gibb's dividing surface". In my opinion, the minimum must coincide with the dividing surface and here it is slightly shifted toward positive values. This may be the consequence of non-equilibrium conditions. Please provide numerical evidence that this is not the case.

Response 1

We disagree with the reviewer that the minimum of the free energy for N_2O_5 must coincide directly with the Gibbs dividing surface, and know of no simple thermodynamic reason this has to be true. For concreteness, we remind the reviewer that the z value we use for N_2O_5 is its center of mass. As N2O5 is not a spherical molecule, it can thus adopt distinct favored orientations at the liquid-vapor interface. Different orientations would result in different positions of the center of mass and thus different positions relative to the Gibbs dividing surface.

More generally, we could artificially change the location of the free energy minimum by employing heavier or lighter isotopes of N_2O_5. As the mass distribution is thermodynamically uncorrelated with the configurational part of the partition function, which determined the free energy profile, this change would alter only the definition of z. We could thus change the minimum location to coincide with the typical locations of any one of the atoms on N_2O_5 without changing any observable thermodynamic properties.

As far as numerical evidence for equilibration, we have included the raw histograms from the umbrella sampling simulations in the SI indicating sufficient overlap to accurately determine the free energy differences between windows.

Comment 2

2) P. 3. Concerning the calculation of Henry's constant and other thermodynamics parameters, I wonder whether these results may be strongly affected by finite size problems. Please comment on that and provide evidence that your simulation box and conditions are accounting for FS effects. In the supp. Material (P. 4) it is reported that the diffusion coefficient is similar to a previous calculation with similar box size. Maybe a shorter simulation of some other parameter relevant to this work with a larger box may support your choice.

Response 2

We thank the reviewer for their question. We expect that the diffusion coefficient is the property that converges the slowest to the thermodynamic limit due to the fact that its finite size effects are dictated by hydrodynamic correlations that decay as $1/L$. This is why we focus on its corrections. Finite size effects that determine thermodynamic properties like the Henry's law constant are expected to decay as $1/L^3$ with a constant of proportionality that is a typical correlation length, which for a liquid is on the order of the molecular diameter. Indeed this has been demonstrated recently for N_2O_5 [3]. N_2O_5 has a diameter roughly equal to 5 Å and the simulation box size we use has a lateral area of 25×25 Å^2, thus we expect corrections to the solubility to be on the order of $(5/25)^3 \approx 1\%$, which is much smaller than the statistical errors we report in the estimate of the Henry's law constant.

[3] Li, W., Pak, C. Y., Tse, Y.-L. S. "Free energy study of H_2O, N_2O_5, SO_2, and O_3 gas sorption by water droplets/slabs." *The Journal of Chemical Physics* **148**, 164706 (2018).

We have revised the manuscript to make this point directly. Specifically we have added a sentence at line 423 that reads "For this slab size, finite size corrections to thermodynamic properties are expected to be

minimal[51].”
